# Validating a point-of-care test for *Toxoplasma gondii* infection in southern sea otters (*Enhydra lutris nereis*)

**DOI:** 10.1007/s00436-026-08725-y

**Published:** 2026-07-07

**Authors:** Ellis Song, Devinn Sinnott, Cara L. Field, Michael Murray, Pádraig Duignan, Melissa Miller, Karen Shapiro

**Affiliations:** 1 Department of Pathology, Microbiology, and Immunology, School of Veterinary Medicine, University of California, Davis, 1 Shields Avenue, Davis, CA 95616 USA; 2 The Marine Mammal Center, 2000 Bunker Road, Sausalito, CA 94965 USA; 3 Monterey Bay Aquarium, 866 Cannery Row, Monterey, CA 93940 USA; 4 California Department of Fish and Wildlife, Marine Wildlife Veterinary Care and Research Center, 151 McAllister Way, Santa Cruz, CA 95060 USA

**Keywords:** Antibody, Detection, Immunochromatographic assay, Point-of-care test, Protozoa, Serology

## Abstract

*Toxoplasma gondii* is a zoonotic protozoan parasite that infects a high proportion of threatened southern sea otters (*Enhydra lutris nereis*) and is an important cause of mortality in this host species. Recently, a point-of-care rapid antibody test (POCT) for *T. gondii* infection was developed for detection of IgG and IgM antibodies in human sera. We aimed to validate the POCT using southern sea otter sera against a gold standard state of infection, based on histopathology, immunohistochemistry, parasite isolation, and PCR. In this study, we hypothesized that the POCT rapid screening tool would offer both high sensitivity and specificity (> 90%) for screening *T. gondii* infection in archived serum samples from southern sea otters with known *T. gondii* infection status. We applied the POCT assay to sera from 109 sea otters (49 negative, 60 positive), and this assay demonstrated an overall sensitivity of 93.3% and specificity of 93.9%. These results indicate that the POCT may be a useful screening tool for *T. gondii* exposure in sea otters. Utilization of this test in wildlife rehabilitation centers would allow for rapid, on-site, and cost-efficient screening of *T. gondii* exposure that could aid in the diagnosis and clinical management of sea otters with suspected toxoplasmosis. Future efforts could target POCT validation in species such as Hawaiian monk seals and Hector’s dolphins, for which *T. gondii is* listed as a threat to species survival.

**Supplementary Information:**

The online version contains supplementary material available at https://doi.org/10.1007/s00436-026-08725-y.

## Introduction

*Toxoplasma gondii* is a zoonotic, protozoan parasite that can infect all warm-blooded animals, including birds, humans, and other mammals. In the environment, this parasite is transmitted via environmentally resistant oocysts shed in the feces of definitive hosts (wild and domestic felids) which can contaminate food and water ingested by humans and other susceptible intermediate hosts (Dubey 2016; Shapiro et al. 2019a). Transmission of *T. gondii* in threatened southern sea otters (*Enhydra lutris nereis*) has been associated with the consumption of benthic invertebrates, such as snails (Johnson et al. 2009), which can concentrate oocysts in their tissues from contaminated seawater (Krusor et al. 2015). Once infective oocysts are ingested by an intermediate host such as sea otters, rapidly multiplying *T. gondii* tachyzoites spread systemically in tissues. Clinical toxoplasmosis in sea otters is typically characterized by meningoencephalitis with severe neurologic signs including seizures, tremors, ataxia, paralysis, and death (Miller et al. 2018). In response to host immune defenses, *T. gondii* tachyzoites transform into slowly replicating bradyzoites that form tissue cysts in the central nervous system, heart, and skeletal muscles and can cause recurrent, chronic clinical disease (Miller et al. 2018).

With an estimated seroprevalence of 57%, infection with *T. gondii* is widespread in southern sea otters (SSO) (Miller et al. 2020) and mortalities can occur following both acute and chronic infections (Kreuder et al. 2003; Miller et al. 2023). The highly virulent COUG strain of *T. gondii* was implicated in SSO mortalities in 2020–2024, causing death in otherwise healthy animals due to severe protozoa-associated steatitis and systemic toxoplasmosis or more classic meningoencephalitis (Miller et al. 2023; Sinnott et al. 2025). This *T. gondii* strain and its associated steatitis syndrome was not observed in this host prior to 2020, and the cause of its emergence in this species remains unknown. The discovery of this highly virulent parasite strain and the importance of toxoplasmosis as a cause of death for SSOs underscores the need for a rapid, cost-efficient test that can be performed on-site at rehabilitation centers to expedite diagnosis and treatment. Currently, the only validated serological test for *T. gondii* exposure in SSOs is the indirect fluorescent antibody test (IFAT) (Miller et al. 2002). The need to ship sera to a specialty lab where the IFAT is performed can create a delay of several days or longer between the time blood is drawn and receiving results, potentially delaying clinicians from initiating timely appropriate antiprotozoal treatment.

In Europe, an immunochromatographic (ICT) antibody point-of-care test designed to detect both IgG and IgM anti-*Toxoplasma* antibodies has been recently approved for rapid exposure detection in humans (*Toxoplasma* ICT IgG-IgM^®^, LDBIO Diagnostics, Lyon, France, hereafter referred to as point-of-care-*Toxoplasma*, or POCT) (Khan and Noordin 2020). Previous reports of application of the POCT in veterinary species include studies conducted on black bears, ring-billed gulls, and black vultures, where the utility of this assay for screening wildlife for *T. gondii* exposure was compared with another serologic test, the modified agglutination test (MAT). However, animals with molecularly and/or histopathologically confirmed positive or negative *T. gondii* infection status were not available to definitively validate the POCT in these hosts (Strules et al. 2023; Rosypal von Dohlen et al. 2024). This lack of ancillary test confirmation of infection status can make it difficult to evaluate and interpret results of real-life applications of the POCT, highlighting the need for validation against a gold standard state of infection.

While the POCT is not able to discriminate between IgM and IgG antibodies, it can yield positive results for acute infections (in which IgM antibodies would be present before IgG antibodies are produced). For these acute cases, conventional testing methods that typically rely only on measuring IgG antibody levels (IFAT and Enzyme-Linked Immunosorbent Assay, or ELISA) may yield false negative results (Khan and Noordin 2020). The POCT is also cost and time efficient compared to other serological tests, which can be limiting factors to optimal diagnostics and care in a wildlife rehabilitation setting. Considering the potential benefits of the POCT, we aimed to evaluate the performance of this test using serum from SSOs that are commonly infected with *T. gondii* and may require veterinary care in rehabilitation centers. We hypothesized that the POCT will offer both high sensitivity (> 90%) and specificity (> 90%) for diagnosing *T. gondii* infection when used to evaluate serum from SSOs with confirmed *T. gondii* infection status.

## Methods

In collaboration with The Marine Mammal Center (TMMC), Monterey Bay Aquarium (MBA), and the California Department of Fish and Wildlife (CDFW), we selected *T. gondii-*infected and uninfected SSOs that stranded along the California coast between 1999 and 2025 for which cryoarchived premortem or perimortem sera was available. Individuals were selected based on records of previous diagnostic testing to identify confirmed *T. gondii* infected and uninfected SSOs. Positive cases were defined as animals with histopathological identification of protozoal organisms in tissues and confirmation of parasite identity as *T. gondii* by immunohistochemistry, isolation of *T. gondii* using cell culture, and/or polymerase chain reaction (PCR) followed by sequencing (Shapiro et al. 2019b; Miller et al. 2020) in at least one tissue (brain +/- heart or skeletal muscle). Negative animals (uninfected controls) were defined as SSOs with no histopathologic evidence of *T. gondii* infection and no amplification of *T. gondii* DNA in three independent biological replicates of at least one tissue (brain +/- heart or skeletal muscle).

**Fig. 1:**
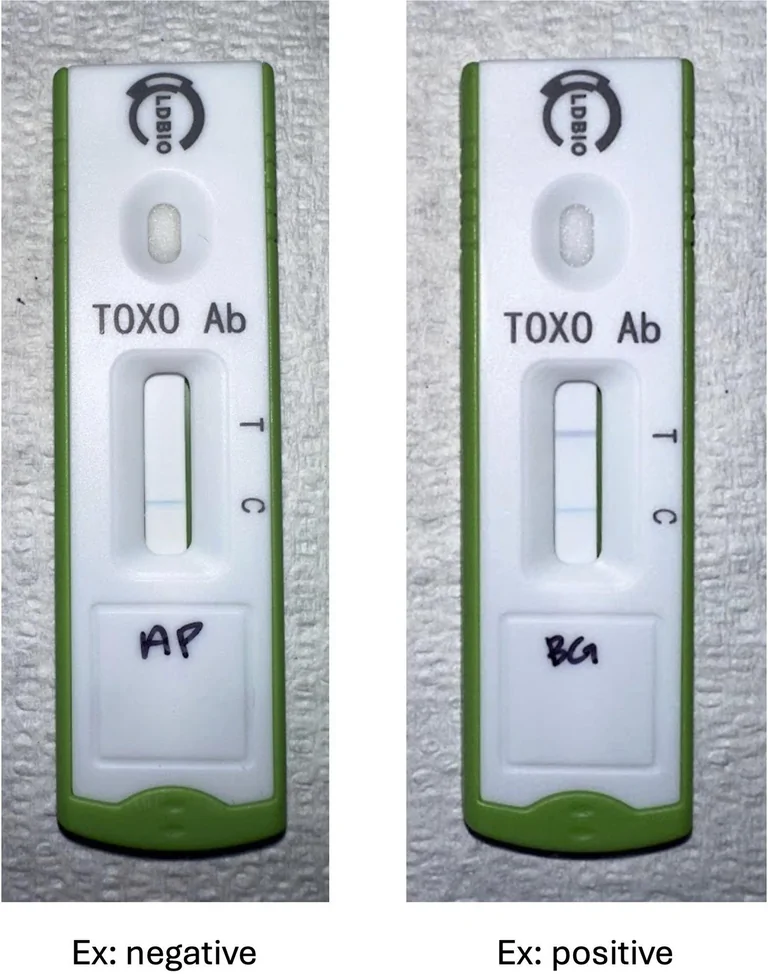
Examples of a negative (left) and positive (right) test results using the ICT IgG-IgM^®^ point-of-care-*Toxoplasma* (POCT) test. The blue line at the “C” mark indicates the control and the presence or absence of the black line at the “T” mark indicates a positive or negative result for the detection of anti-*T. gondii* antibodies

Following manufacturer’s instructions, we performed the POCT on SSO sera from the selected cases and controls. Briefly, 15 ul of serum was applied to a well in the lateral flow cassette followed by 4 drops of eluent, per manufacturer instructions. The results were recorded after 30 min as positive (black line at the ‘T’ (test) mark and blue line at the ‘C’ (control) mark) or negative (no line at the ‘T’ mark, blue line at the ‘C’ mark (Fig. 1). Samples for which a control line was not visible were repeated. To evaluate the performance of the test, we calculated the sensitivity, specificity, positive predictive value, and negative predictive value (Trevethan 2017).

**Fig. 2:**
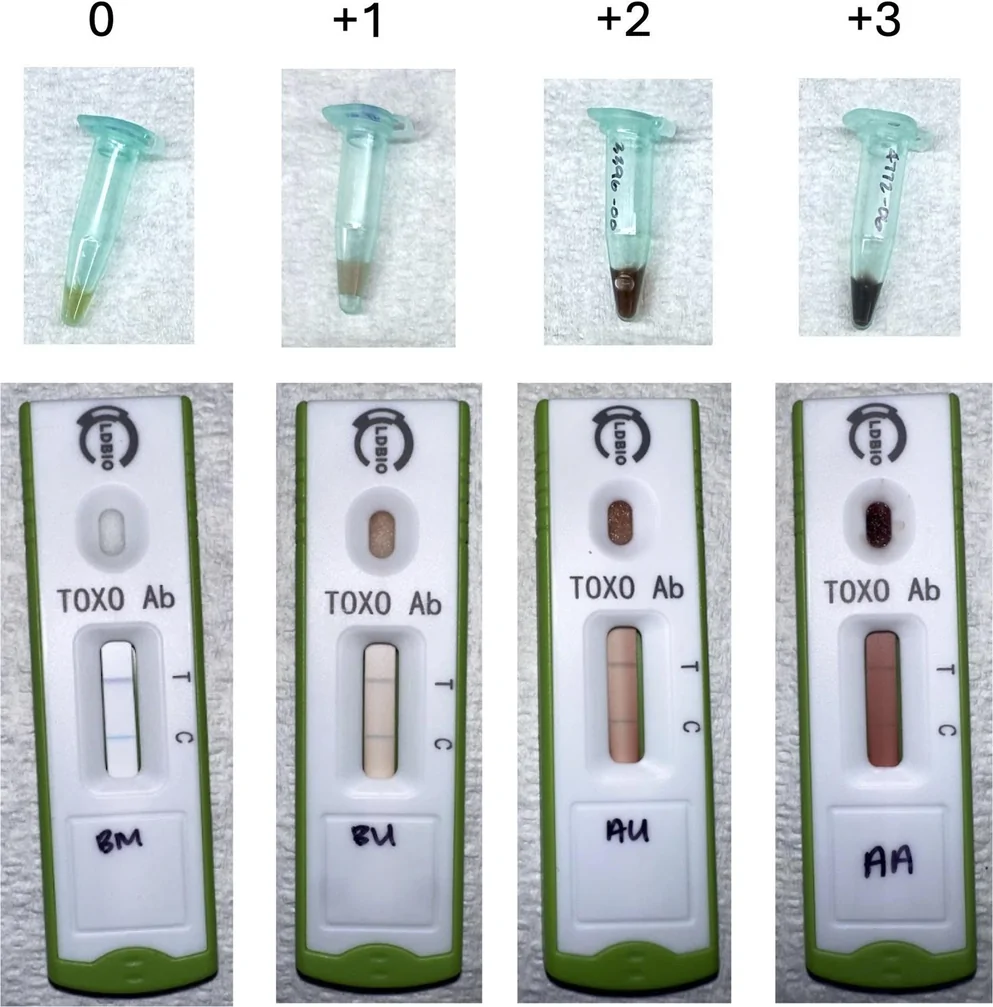
Examples of varying levels of hemolysis (scored as 0, +1, +2, +3) of the serum (top) and the assay cassette window (bottom). Despite the reddish background for hemolyzed samples on the test cassette, a positive line could still be discerned for SSO infected with *Toxoplasma gondii*

In the test manual, the manufacturer advises against the use of hemolyzed sera as hemolysis can make interpretation of test results challenging; however, the manual also states that there is no evidence of hemolysis influencing the performance of the test itself. Obtaining serum from wildlife species, including SSOs, can often be difficult and can lead to hemolyzed samples. To further evaluate the effect of serum hemolysis on test accuracy, we recorded the degree of hemolysis for both the serum itself and how the assay cassette appeared once each sample was applied to the test strip (Fig. 2). The degrees of hemolysis for the tubes and test strips were scored separately on a scale from 0 (no hemolysis) to + 3 (severely hemolyzed). The test manufacturer also recommends using either fresh serum or samples that were frozen for two years or less for this test. However, cryoarchived samples exceeding this recommended two-year limit were needed in order to achieve an adequate sample size to validate the POCT for use in SSOs. The minimum sample size required to calculate our predicted sensitivity (90%) and specificity (90%) with 95% confidence intervals and 80% power was *N* = 70 (35 positive and 35 negative SSOs) (Hajian-Tilaki 2014).

Performance of the POCT was assessed by calculating sensitivity, specificity, and positive and negative predictive values:

$$\mathrm{Sensitivity}\:\mathrm{(Se)}=\:\frac{a}{a+c}\times100$$

$$\mathrm{Specificity}\:\mathrm{(Sp)}=\:\frac{d}{b+d}\times100$$

$$\mathrm{Positive\:predictive\:values}\:\mathrm{(PPV)}=\:\frac{a}{a+b}\times100$$

$$\mathrm{Negative\:predictive\:values}\:\mathrm{(NPV)}=\:\frac{d}{c+d}\times100$$

Where “a” is true positives, “b” is false positives, “c” is false negatives, and “d” is true negatives.

To evaluate the effects of hemolysis and sample age of serum on test accuracy, we applied the Fisher’s exact test using Stata (StataCorp, 2025). Significance was determined at *P ≤* 0.05.

## Results and discussion

Based on our selection criteria and the previously performed gold-standard diagnostic results available, we identified 60 SSO with histopathologic, culture, and/or PCR-confirmed *T. gondii* infection and 49 negative controls for a total sample size of 109. A positive POCT result was obtained for 56 of the 60 SSO with confirmed *T. gondii* infection, yielding a test sensitivity of 93.3%. Of the 49 negative controls, a positive POCT result was recorded for three animals (false positives) yielding a specificity of 93.9%. The positive and negative predictive values were calculated as 94.9 and 92.0%, respectively (Table 1).

**Table 1:** Sensitivity and specificity of *Toxoplasma gondii* point-of-care test (POCT) compared with a gold standard of confirmation *T. gondii* infection or non-infection based on histopathology, immunohistochemistry, culture, and/or PCR in SSOs (*Enhydra lutris nereis*) from California.

POCT test result	*T. gondii* positive cases	*T. gondii* negative cases	Totals	
Positive	56	3	**59**	**PPV = 94.9%**
Negative	4	46	**50**	**NPV = 92.0%**
Totals	**60**	**49**	**109**	
	**Se = 93.3%**	**Sp = 93.9%**		

*Se *sensitivity, *Sp *specificity, *PPV *positive predictive value, *NPV *negative predictive value

We evaluated the association between test performance and the level of hemolysis, both for the serum directly and its appearance on the test strip (Fig. 2), using a Fisher’s exact test. The level of hemolysis was analyzed as both a categorical variable ranging from no, mild, moderate to marked hemolysis (denoted as 0, + 1, +2, and + 3, respectively) and as a binomial variable (unhemolyzed vs. hemolyzed). No significant association was observed between test performance (correct classification of infection status) and level of serum hemolysis in categorical analyses (*p* = 0.117). Overall, no negative effects of hemolysis were observed on test accuracy, when analyzing both the hemolysis score of the sera directly or discoloration of the test cassette, using either binary or categorical classification of hemolysis.

We also evaluated whether serum age affected test performance, defining accuracy as the ability of the assay to correctly determine infection status compared with the gold standard. We categorized the age of serum as a binomial variable (older than two years vs. younger than two years) based on the year of collection and the year the POCT was performed. No significant association was observed between serum age and test accuracy (Fisher’s exact test, *p* = 0.211).

Our study validates the POCT for use in SSOs using molecular and histopathologic confirmation as the gold standard state of infection. Previous studies in other veterinary species have evaluated the POCT in comparison to another serological test (Strules et al. 2023; Rosypal von Dohlen et al. 2024). Use of a molecular and histopathology-based gold standard more closely reflects the true state of infection and has enabled us to more precisely evaluate the sensitivity and specificity of the POCT in SSOs and assess its potential utility as an antemortem and postmortem diagnostic test for *T. gondii* infection in this species. Our results revealed that the POCT has a high sensitivity (93.3%) and specificity (93.9%) for detecting anti-*T. gondii* antibodies in SSO serum, making it an effective tool for rehabilitators to rapidly screen for *T. gondii* infection. This is a more conservative estimate than its performance in humans (100% sensitivity and 97.7% specificity per manufacturer’s records).

By evaluating the POCT against molecular and histopathological evidence of infection rather than an alternative serological test, we intended to evaluate POCT performance relative to evidence of infection rather than relative agreement with an existing serologic platform. This method limits bias estimates of diagnostic sensitivity and specificity when measuring biologic processes using serologic assays (Gardner et al. 2000) and is consistent with recommendations outlined by the World Organization for Animal Health (WOAH, 2023; formerly OIE). However, when interpreting sensitivity and specificity in light of this reference standard, we must also acknowledge that PCR and histopathology have their limitations: both PCR and histopathology assess small sections of tissue that can potentially miss parasites that are randomly or sparsely distributed within tissue, even if multiple replicates are evaluated. This may result in apparent POCT false positive cases, as some chronically infected animals with low parasite burdens may have been misclassified as uninfected by PCR and histopathology.

To date, the only validated serological test for assessing *T. gondii* infection in SSO is the IFAT (Miller et al. 2002). The sensitivity and specificity of the IFAT for detecting anti-*T. gondii* antibodies in SSO serum were previously evaluated to be 96.4% and 67.3%, respectively (Miller et al. 2002). The case definitions, gold standard criteria, and samples used for validation of the IFAT assay differed in criteria used in this study; therefore, a true comparison of test performance between the IFAT and POCT cannot be drawn. While the POCT provides a time- and cost-efficient screening test for assessing *T. gondii* exposure, the IFAT can provide additional clinically valuable information such as a semi-quantitative titer for *T. gondii* antibodies, and can be run in parallel to detect *Sarcocystis neurona* antibodies, another closely related protozoan parasite that commonly infects SSOs and can cause a similar clinical presentation. The POCT may serve as a rapid benchtop screening tool for veterinary staff in marine mammal rehabilitation facilities to help triage animals, expedite screening for *T. gondii* infection, and guide treatment. Because the effect of serum hemolysis was found to be negligible, the POCT might also be a helpful benchtop screening tool for necropsied SSOs as well.

Our study was limited by the serum samples available from SSOs with corresponding histopathological data and tissues for molecular diagnostics. The samples used in this study were cryoarchived between < 1 and 25 years since the initial date of collection, and many samples had been previously frozen and thawed before use. It is possible that these factors may have caused some degree of antibody degradation in some serum samples which could have influenced the results of the POCT. Our analysis revealed that there was no significant difference in test accuracy when using serum greater than or less than the two-year cut-off that was recommended in the manufacturer’s instructions. However, continued evaluation of the POCT using freshly obtained serum may yield higher sensitivity and specificity estimates. The samples used in this study also had varying degrees of hemolysis. While non-hemolyzed sera is preferred by the manufacturer for use in humans, providing veterinary care for wildlife in rehabilitation centers presents a unique set of challenges that may unavoidably result in the collection of hemolyzed serum. While our results suggest that hemolysis does not adversely affect the test accuracy, it is important for veterinary staff to exercise caution when using this test with hemolyzed sera.

Several considerations should be taken into account when interpreting our study’s findings. First, given the high prevalence of infection of both *T. gondii* and *S. neurona* in SSOs, potential cross-reactivity between these protozoal parasites is an important consideration. Due to logistical and cost constraints, we were unable to identify and test sera from a substantial number of SSOs with confirmed *S. neurona* infections and without evidence of *T. gondii* infection. In a pilot sample of eight SSOs with confirmed *S. neurona* infection and without molecular evidence of concurrent *T. gondii* infection, the POCT results were negative for 7 of these 8 animals (Table S2). Interpretation of POCT results in the context of clinical toxoplasmosis versus chronic subclinical infection is also relevant for rehabilitation and future surveillance applications. The methods used to classify true infection status (parasite isolation, histology, and PCR) can identify both clinical toxoplasmosis and latent (subclinical) infections (Miller et al. 2020; Shapiro et al. 2019b) We were able to identify the cause of death (COD) for 45 *T. gondii-*infected SSOs in our study. Of these 45 animals, *T. gondii* was determined to be a primary or contributing COD for 19 SSOs, while 26 had incidental infections unrelated to the identified COD (Table S1). This suggests that the POCT can detect anti-*T. gondii* antibodies in both clinical and incidental cases of *T. gondii* infection. Finally, because *T. gondii* is fairly prevalent in SSOs, the predictive values reported here may differ in populations with different infection prevalences.

Considering the many differential diagnoses for SSOs that strand with neurological clinical signs, including protozoal infections (*T. gondii* and *S. neurona*) (Miller et al. 2018), domoic acid toxicosis (Miller et al. 2021), morbilliviruses (Thomas et al. 2020), and trauma, establishing a rapid and accurate diagnosis for these similarly presenting conditions is often challenging. However, wildlife veterinarians and rehabilitation professionals must respond quickly to implement appropriate treatment that can reduce morbidity and mortality. Our study demonstrates that the POCT is a highly sensitive and specific test for screening SSO sera for *T. gondii* infection and can provide rapid, onsite results to rule out *T. gondii* infection or determine the need for additional diagnostics when managing SSOs in rehabilitation facilities. Future application of the POCT in other *T. gondii* susceptible wildlife would provide a valuable clinical screening tool for these vulnerable marine mammal species, such as Hawaiian monk seals (*Monachus schauinslandi*) and Hector’s dolphins (*Cephalorhynchus hectori*) for which *T. gondii* is listed as a threat to species survival (Roe et al. 2013; Robinson et al. 2023). 

## Supplementary information

Below is the link to the electronic supplementary material.

Supplementary Material 1

Supplementary Material 2

## Data Availability

All data supporting the findings of this study are available within the paper and its Supplementary Information.

## Declarations

CDFW handled stranded sea otters under Sect.  109(h) of the U.S. Marine Mammal Protection Act (MMPA), in accordance with U.S. Fish and Wildlife Service (USFWS) regulations implementing the MMPA (50 CFR 18.22[a]) and the U.S. Endangered Species Act (50 CFR 17.21[c][3]). TMMC and MBA conducted animal recovery, rehabilitation, humane euthanasia, and sample collection under USFWS permits MA101713-1 and PER4324646 (TMMC), and MA032027 and MA186914 (MBA), respectively. These statutes and permits authorize wildlife agencies and rehabilitation centers to intervene on behalf of free-ranging marine mammals for their protection and welfare, including rehabilitation and humane euthanasia when indicated. All animal handling and euthanasia procedures were conducted under standardized institutional animal care and veterinary oversight protocols, and gross necropsies were performed on all sea otters by veterinary pathologists and trained staff at CDFW and TMMC. Serum and other biological samples were collected from fresh or previously frozen/thawed carcasses during postmortem examination.
